# BFPTool: a software tool for analysis of Biomembrane Force Probe experiments

**DOI:** 10.1186/s13628-016-0033-2

**Published:** 2017-02-13

**Authors:** Daniel Šmít, Coralie Fouquet, Mohamed Doulazmi, Frédéric Pincet, Alain Trembleau, Martin Zapotocky

**Affiliations:** 1 Institute of Physiology, Czech Academy of Sciences, Vídeňská 1083, Prague, 14220 Czech Republic; 2 Institute of Biophysics and Informatics, First Faculty of Medicine, Charles University in Prague, Kateřinská 2, Prague, 12000 Czech Republic; 3 Sorbonne Université, UPMC Univ Paris 06, INSERM, CNRS, Neurosciences Paris Seine – Institut de Biologie Paris Seine (NPS – IBPS), 9 Quai Saint Bernard, Paris, 75005 France; 4 Sorbonne Université, UPMC Univ Paris 06, CNRS, Biological Adaptation and Ageing – Institut de Biologie Paris Seine (B2A – IBPS), 7 Quai Saint Bernard, Paris, 75005 France; 5 Laboratoire de Physique Statistique, École Normale Supérieure, PSL Research University, Paris, France; 6 Université Paris Diderot Sorbonne Paris Cité, Paris, France; 7 Sorbonne Universités UPMC Univ Paris 06, CNRS, 24 rue Lhomond, Paris, 75005 France

**Keywords:** Biomembrane Force Probe, Motion tracking, Image analysis, Force spectroscopy, Cell mechanics

## Abstract

**Background:**

The Biomembrane Force Probe is an approachable experimental technique commonly used for single-molecule force spectroscopy and experiments on biological interfaces. The technique operates in the range of forces from 0.1 pN to 1000 pN. Experiments are typically repeated many times, conditions are often not optimal, the captured video can be unstable and lose focus; this makes efficient analysis challenging, while out-of-the-box non-proprietary solutions are not freely available.

**Results:**

This dedicated tool was developed to integrate and simplify the image processing and analysis of videomicroscopy recordings from BFP experiments. A novel processing feature, allowing the tracking of the pipette, was incorporated to address a limitation of preceding methods. Emphasis was placed on versatility and comprehensible user interface implemented in a graphical form.

**Conclusions:**

An integrated analytical tool was implemented to provide a faster, simpler and more convenient way to process and analyse BFP experiments.

**Electronic supplementary material:**

The online version of this article (doi:10.1186/s13628-016-0033-2) contains supplementary material, which is available to authorized users.

## Background

With the advent of modern biophysical techniques and devices [1, 2], the need for image analysis (IA) of the recordings and automatic processing of obtained data has been growing. Versatile IA open software solutions, e.g. ImageJ [3], Icy [4], Vaa3d [5] and cellProfiler [6], were developed to provide the broad scientific community with tools for analysis of general experimental data. Open-source developers and research groups involved in those software projects contributed many powerful and accessible plug-ins for widely used technologies and common IA tasks. In addition, a plethora of specialized algorithms were designed to process the primary image data of particular experimental devices. The specialized algorithms are however typically complex, their implementation often lacks a graphical user interface (GUI) and is seldom publicly available or documented. Therefore, user-friendly dedicated IT tools are still lacking for specific biophysical techniques; one of such techniques is the Biomembrane Force Probe (BFP). This technique could become more accessible if robust post-experiment analysis were readily feasible—it would decrease the need for specialized equipment, relax constrains on experimental procedure and imaging quality, and allow repeated and more sophisticated analysis. Due to technical constraints (limitations on the bandwidth of video recording and on data file size), such an approach was not practical in the early days of the BFP technique (1990s), but has become readily feasible with current standard equipment. The aim of this work is to provide a Matlab-based processing and analytical tool with intuitive GUI precisely for the BFP.

**Fig. 1:**
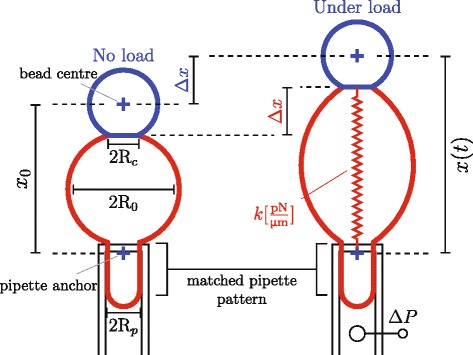
Probe calibration illustration. In the linear elastic regime, the probe can be described as a spring with stiffness *k*. The stiffness depends on geometry (*R*_0_,*R*_*c*_,*R*_*p*_) of the probe and the aspiration pressure *Δ* *P* and is given by Eq. 1. The force at any time is given by the deformation of the RBC *Δ* *x*(*t*)=*x*(*t*)−*x*_0_, where *x*_0_ is a reference distance measured when the RBC is not under load. It is effectively measured as the distance between the centre of bead and a particular anchor point on the pipette (marked by *blue crosses*)—a change in this distance corresponds to the change in length of RBC, *Δ* *x*

The BFP technique (experimental configuration illustrated in Fig. 1) was invented by Evans et al. [7] as a suitable tool for single-molecule force spectroscopy [8] and experiments on biological interfaces [9]. While the purpose of single-molecule force spectroscopy is to determine the single bond energy landscape, lifetime and failure probability under particular load and loading rate [10], experiments on biological interfaces focus on surface adhesion energy and membrane rigidity. The BFP can be directly used in culture medium, it interacts with cells softly and allows measuring a wide range of forces, from tenths of piconewton to nanonewton, e.g. to record the breaking of a protein bond [11–13]. It is composed of a micro-pipette connected to a controller acting as a manipulative apparatus, and of a red blood cell (RBC) acting as a force transducer. The RBC is aspirated in a pipette and a bead is attached to its apex to serve as an intermediary element. Typically, a biotinylated RBC and a streptavidin-coated bead are used; when brought in contact, a streptavidin-biotin link is formed, keeping the bead attached to the RBC. The bead can then form another bond of interest with a single molecule (e.g. protein, DNA) or with another interface (e.g. cell, vesicle). Controlled movement of the RBC-mounted pipette (RMP) or of the studied sample imposes a load *F* on the studied bond/interface, mediated by the bead and transduced by the RBC. The RBC undergoes a deformation proportional to the load, *Δ* *x*∝*F*. The deformation *Δ* *x* can be measured by tracking the centre of the circular bead and a specific point on the pipette tip; the change in the distance of these two tracked points corresponds to the RBC deformation.

Surprisingly, no freely-available dedicated processing software is available to analyse BFP recordings. In [11, 14], the bead tracking was performed in real time during the experiment using custom software, fitting a Gaussian blob onto the bead image intensity profile. An alternative method was developed in the proprietary software LabView [15], detecting the bead fringe as a minimum in contrast along the probe axis. To our knowledge, none of these software solutions were publicly released. These methods were implemented for experiments in which the RMP was not tracked (i.e. either remained static, or had position obtained indirectly from piezo controller movements), the field was clear and the focus reliable. This would not be the case for many biological applications; the user might need to move the RMP freely, will be faced with debris, unstable focus, littered field and constrains that prohibit the movement of the working stage. Under such circumstances, it is useful to have a more versatile tool offering not only bead tracking but also pipette detection, adjustments, exceptions treatment, thresholding, adaptation and basic analytical features.

The present software tool addresses these points. It provides a GUI to keep all the functions easily accessible to the end-user, while their implementation is contained in a single, easily installable package. Importantly, it is designed to analyse the captured videos after the experiment, allowing the user to make necessary or optional adjustments to the processing of the recording. The tool is written in Matlab, which makes it cross-platform accessible, and an extensive I/O interface is implemented to allow for post-processing in other programs and easy transferability of the results.

## Implementation

The tool is presented in the form of a Matlab application package, bundled into an application installation file **BFPTool.mlappinstall**. It can be easily installed into the Matlab application dashbar and run by clicking the icon. The application was tested in Matlab version 2015b and 2016a with Image Processing Toolbox, under Linux (Ubuntu 16.04), Windows 10, and Mac OS 10.9 and later. It is accompanied with a user guide and essential documentation, which are embedded in the installation file; after the first run of the application, the documentation can be reached directly from the home page in the Matlab help browser, and is fully searchable for keywords. During the application installation, the source M files are unpacked into Matlab application folder; the code has been commented extensively and can be readily modified by users to suit their own needs. For convenience of the user, the application was designed for two distinct scenarios of analysis. A basic scenario, using default settings, is suitable for good quality recordings, allowing to do fast tracking and to immediately plot the measured deformation *Δ* *x*. The user quickly obtains preliminary qualitative information about the force time course, without a need to study the program interface and functions. The second scenario is more detailed, and allows the user to adjust tracking intervals and parameters, visualize the tracking results in different ways, precisely measure the parameters of the probe geometry, and perform basic analysis of the time course of the calculated force and fidelity metrics.

### Computational functions

The main purpose of the application is to calculate the applied force time course, *F*(*t*). This is implemented by the Matlab class *BFPClass* (child of the native *handle* superclass). The instances of this class are constructed with initial information about the experiment (e.g. geometry, pipette and bead tracking settings, list of frames for processing), they store results (e.g. bead/pipette coordinates, log of bad frames, force) and perform some of the complementary tasks (e.g. plotting, stiffness calculation). The class calls two external functions to perform the tracking of the bead and the pipette tip and calculates the force based on the returned results.

We assume that the system is approximately axial (centres of the bead and RBC, and the pipette anchor, are all on one axis), so the extension or compression of the RBC along the axis, *Δ* *x*(*t*), is equal to the change in distance *x*(*t*) between the bead centre and the pipette tip anchor, compared to such distance when the RBC is under zero load, *x*_0_ (see Fig. 1). The positions of the bead centre and the pipette tip anchor at the given time therefore determine the RBC deformation and consequently the force, *F*(*t*).

The bead tracking algorithm is based on Matlab’s circle detection function *imfindcircles* from the Image Processing Toolbox, which implements a circular Hough transform, either two-stage or phase coding method. Both algorithms are applied on each frame, on the area around the last confirmed presence of the tracked bead. A list of potential candidates is built from scores (above a threshold) based on the *imfindcircles* detection metric *M*_*b*,*i*_ (where the index *i* numbers the candidates) and the distance *d*_*i*_ of candidate centre from the centre of the last known position of the bead, with candidates’ radii within the range (*R*_<_,*R*_>_). The bead metric is the value of the accumulator array for the given pixel returned by the *imfindcircles* method, usually in the range *M*_*b*,*i*_∈(0,3). A distance factor $f_{i}=\frac {\max (d_{i},R_{<})}{R_{<}}$ is used to decreases the weight of candidates that moved too far; the final sorting scores are $\frac {M_{b,i}}{f_{i}}=\frac {M_{b,i}R_{<}}{\max (d_{i},R_{<})}$. The highest score, $M_{b}=\max \left (\!\frac {M_{b,i}}{f_{i}}\!\right)$, is selected as the match, the position is logged and used to calculate *d*_*i*_ for the next time frame. The centre of the bead is detected with sub-pixel precision, based on the accumulator array result. The major advantage of this method is that it detects also partially obscured objects, however the more edge pixels are obscured the lower the precision is. The detection accuracy depends on the bead size and image quality, and is typically in the range 30-50 nm.

The pipette tracking algorithm uses pattern matching, based on evaluating the normalized cross correlation (Matlab *normxcorr2* function) of a user-delineated template of the pipette tip with sub-frames of the video. After the template is repeatedly matched, being moved pixel by pixel across a restricted area in the video frame, the correlation coefficients (for each template position), are saved in a 2D array, *M*_*p*,*ij*_. The best match, *M*_*p*_= max(*M*_*p*,*ij*_), is determined at single-pixel precision, and the value *M*_*p*_ represents the pipette tracking metric value for the given time frame. The array is then interpolated by an elliptical paraboloid to determine the coordinate of maximal correlation with sub-pixel precision, typically $\lesssim $10 nm—the detection is more precise than the detection of the bead. The pipette detection is generally very robust and stable during the recorded time course. The procedure implements additional steps, like dilation (erosion) of the pattern, in case the correlation score becomes too low. In such case, the initial frame of the processed time interval is tested against the current pattern. If the pattern is reliably localized, a set of new patterns is extracted from the initial frame by delimiting larger or smaller areas (dilation or erosion). Such new patterns are matched in the problematic frame of low metric reading (which evoked the correction procedure); the final pipette location in the frame is determined by the pattern yielding the highest correlation metric score.

The pattern matching is a direct method of pipette position tracking with precision $\lesssim $10 nm. It is usable with any pipette manipulation setup, thus permitting the use of micromanipulators with unrestricted direction, range and speed. Setups based on piezo translators allow an even higher precision (around 1 nm) of pipette tracking using the piezo feedback signal; however, such setups have a limited operation range (typically around 15 *μ*m) and offer significantly less flexibility.

**Fig. 2:**
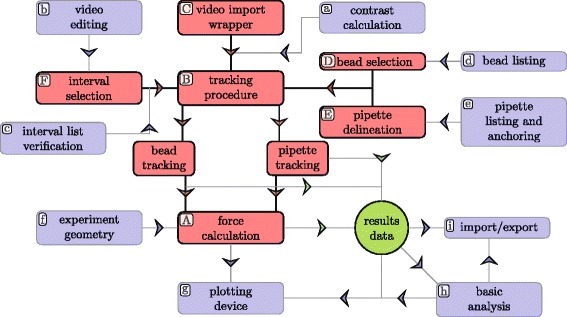
Program workflow. Red nodes (labelled by *upper-case letters*) represent the basic workflow of the program, which requires little user input. The *blue* nodes provide the optional means of adjustment and optimisation (labelled by *lower-case letters*). The nodes are referred to by their labels at the appropriate sections of the text, where more details are provided

The bead and pipette tracking procedures determine the RBC deformation at any given time, *Δ* *x*(*t*)=*x*(*t*)−*x*_0_ (*B* in Fig. 2). The force calculation (*A* in Fig. 2) is performed in linear regime of force-extension relation (see Fig. 1), i.e. *F*=*k*·*Δ* *x*, where *k* is the stiffness of the probe. The stiffness is calculated using the following equation [16, 17]:

1 $${} k = R_{p} \Delta P\frac{\pi}{(1-\hat{R}_{p})} \frac{1}{\log\!\left(\!\frac{4}{\hat{R}_{c}\hat{R}_{p}}\right)- \left(1 - \frac{1}{4} \hat{R}_{p} - \frac{3}{8} \hat{R}_{p}^{2} + \hat{R}_{c}^{2}\right)}  $$

where *R*_*p*_ is the pipette radius, *R*_*c*_ is the contact radius and *R*_0_ is the radius of aspirated RBC without any load; *Δ* *P* is the aspiration pressure (see Fig. 1). The hat designates a radius normalized as fraction of radius of the RBC (*R*_0_), e.g. $\hat {R}_{p}{=}\frac {R_{p}}{R_{0}}$. The linear force-extension regime is known to hold well for RBC deformations smaller than *Δ* *x* < 0.5 *μ*m; when this threshold is exceeded, the linear relation overestimates the force.

If no calibration information is provided, the program will calculate the time course of change in length of the RBC, $\Delta \tilde {x}(t)$ ^1^. This change in length corresponds to the deformation of RBC ($\Delta \tilde {x}{=}\Delta x$) only if the reference length, *x*_0_ (corresponding to unstrained RBC), is properly defined (i.e. $x_{0}{=}\tilde {x}_{0}$)—i.e., the appropriate frame *t*_0_ is selected as illustrated in Fig. 1. The program otherwise extracts an arbitrary reference length $\tilde {x}_{0}$ from the first frame of processed interval, leading to results shifted by $x_{0}-\tilde {x}_{0}$ from the true value. The program also contains predefined generic values for probe geometry and aspiration pressure ($\tilde {R}_{p}$=1.0 *μ*m, $\tilde {R}_{0}$=2.5 *μ*m, $\tilde {R}_{c}$=0.75 *μ*m, $\Delta \tilde {P}$=200 Pa), which can be used to calculate a generic stiffness $\tilde {k}$ and an order of magnitude estimate of the non-calibrated applied force $\tilde {F}$.

The tool however contains a feature to conveniently measure the necessary radii of the probe to obtain *k*. The reference frame with the unstrained RBC, to calibrate zero force, has to be always selected based on user’s judgement.

### Basic analysis

Preliminary analysis of the data can be performed in several direct steps, without setting any particular parameters. Please refer to Fig. 2 to see the functions in context of the workflow; the basic program features form an integrated set, coloured in red and labelled by capital letters.

The analysis begins with loading of a video of the BFP experiment (*C* in Fig. 2). Standard video formats compatible with Matlab are supported (including AVI, MP4, MOV, WMV—a complete list can be found at MathWorks website [18]); Matlab also supports TIFF stacks through LibTIFF library [19]. Note that TIFF metadata fields are not always reliably populated—in case the program cannot detect the framerate parameter, the user is prompted to provide the information during the video import or change it in the GUI. The implemented wrapper class *vidWrap* provides a unified interface for all types of media files; it contains several verification procedures and maintains basic information about the video file and its contrast metric. Once the video file has been opened, GUI buttons are enabled and the user can select a bead to track (*D* in Fig. 2) and delineate a pattern of pipette tip (*E* in Fig. 2) to match. Having chosen the interval (*F* in Fig. 2) of frames to process, the tracking procedure can be started (*B* in Fig. 2), the *BFPClass* object is constructed and the functions invoked. The program tracks the bead and the pipette, one after the other.

Several performance metrics are evaluated to ensure robust tracking. The metrics *M*_*p*_ for pipette and *M*_*b*_ for bead detection were already presented in the section Computational functions. In addition, two types of contrast metric are defined: (1) the time course of the standard deviation (SD2) of pixel intensities in each frame, normalized by the maximal value during the time course and (2) the running standard deviation (rSD2) of SD2 values (based on a 40-frame window by default), also normalized by the maximum. In case the SD2 contrast metric decreases below a threshold (SD2<0.95), or the tracking metrics do (*M*_*p*_<0.95, *M*_*b*_<0.8 by default), the program issues a warning, logs the frame of incident, and attempts to proceed with tracking, unless either pipette or bead cannot be recognized over several consecutive frames (5 by default). In a successful case, tracking across the interval is completed, the deformation (force) time course is plotted and intervals of potential detection instability are reported. If the probe is not calibrated, the method will invoke generic probe geometry and provide the deformation and the uncalibrated force readings.

### Optional features

The tool contains many functions allowing the user to narrow down or optimize the analysis. These are marked by blue nodes and labelled by lower-case letters in Fig. 2. The possibilities are discussed as they follow the main workflow.

Upon video upload, the SD2 contrast metric is calculated and displayed (*a* in Fig. 2), helping to keep track of image quality and to suggest sections of the video possibly unsuitable for analysis. Similarly, the rSD2 metric is shown, to point out intervals of variable contrast, most often resulting from focus shifts. If the value of the SD2 metric changes and remains stable during a well-defined interval, the user may try to track the affected interval separately, using an alternative pipette tip pattern. A continually fluctuating contrast however perturbs the response of pipette pattern-matching and bead detection, which impairs the precision of RBC deformation measurement; such intervals are better excluded.

Using the contrast data or making deliberate choices, the user can take advantage of a video-editing interface (*b* in Fig. 2) to construct a chain of non-contiguous intervals to track. This is a key feature for analysis of suboptimal recordings. Such recordings contain one or more intervals of frames, where the straightforward method proposed in the section Basic analysis fails for reasons as faltering focus, severe obscuring of the bead, physical changes to the probe (e.g. RBC replacement, separation of the probe), or straying out of the field. It allows the user to subdivide the recording analysis, defining a pipette pattern to be shared among some intervals, while changing the inputs for other intervals (*F* in Fig. 2). The interval selection procedure runs a series of verifications and displays informative messages, to ensure all intervals are properly constructed, eventually prompting the user for ad hoc corrections or additional input (*c* in Fig. 2).

The interval chain construction is aided by supplementary functions (*d, e* in Fig. 2), which allow to save a particular bead and pipette pattern and reuse them later. This can be particularly useful when sections of video suitable for processing are heavily fragmented or when the user repeatedly updates the selection of intervals.

The tracking procedure itself can be finely tuned. Most of the thresholds and sensitivities are adjustable. When the program detects under-performing metrics, besides issuing warnings, it adaptively attempts to refine the detection by changing some of these thresholds or loosening restrictions on the detection area.

It is not necessary to set any parameters to obtain uncalibrated deformation and force time courses, as outlined in the Basic analysis section. To obtain a precise reading of force, the probe must be calibrated by assigning the RBC stiffness *k* (see Eq. 1). The geometrical parameters, *R*_*p*_, *R*_*c*_, and *R*_0_ can be conveniently measured directly in the video using the program’s dedicated interactive feature (*f* in Fig. 2). In case the explicit video resolution (pixel to micron ratio) is unknown, it can be calibrated by measuring the video scale bar or any object of known dimensions. As a result, only the aspiration pressure *Δ* *P* in Pascals must be input by the user as an external parameter—everything else can be extracted from the recording itself.

Having performed the tracking, its quality can be directly verified by displaying an overlay marking the detected coordinates over the main video frames in the GUI video player. A red circle delineates the detected bead’s edge and a small blue circle identifies the anchor point on pipette tip. This feature is the most direct conceivable way to assure oneself that the tracking was carried out successfully and accurately. More complementary information is accessible through a graphing panel embedded in the GUI (*g* in Fig. 2). Any obtained data can be visualized: trajectories of the pipette and the bead in time, calculated deformation *Δ* *x*(*t*) and force *F*(*t*), as well as contrast metrics (SD2, rSD2) and tracking metrics (*M*_*b*_, *M*_*p*_).

Some elementary analytical tools were implemented as part of the graphing panel. User can select an interval of interest in the obtained data, and fit the data with a straight line or an exponential, or run a plateau detection procedure (*h* in Fig. 2). These simple regressions can provide initial information about the loading rate, relaxation time of the system, or an average force applied during an equilibrium situation.

Finally, we provide several import/export options (*i* in Fig. 2). It is possible to export graphs, images, data (in CSV format) to a file or to the Matlab base workspace. The major feature is the possibility to save the whole GUI session at the given moment, and load it later to continue the analysis. The corresponding MAT file is fully transferable, and may be imported into the tool runing on another computer (note that the video file must be also present on the target machine).

## Results and discussion

In this section, we will demonstrate both approaches outlined in the previous section, running analyses on a simple example (with a single tracked interval) and on more complex videos. The videos originate from our BFP experiments and represent typical examples.

The experiments were performed on biotinylated olfactory epithelium explants after 2 days of cultivation. Streptavidin-coated 3 *μ*m diameter beads were added to the culture and attached to the biotinylated axons during cultivation. RBCs were obtained from a drop of human blood, drawn with a medical lancing device; laboratory gloves were used while handling the blood to prevent infection risk. The protocol was approved by the ENS Safety and Ethics Committee; informed and written consent to participate was obtained from the healthy volunteer whose drop of blood was taken. The isolated RBCs were stored in phosphate buffered saline. The RBCs were then covalently linked with PEG-biotin polymers (purchased from Interchim), following the protocol kindly provided by E. Evans [20]. The biotinylated RBCs were used for BFP measurements within 3 weeks.

The BFP manipulations were done in a thermostated chamber (37 °C) mounted with the Leica DMIRB inverted microscope, and recorded by a JAI CCD digital camera. Biotinylated RBCs were added to the culture, one was selected and aspirated into the micropipette (of diameter 1.5–2.0 *μ*m, aspiration pressure 200–250 Pa), and brought in contact with a bead to allow a streptavidin-biotin link to be formed. The assembled probe was then slowly manipulated to gradually deflect the attached axon. More context can be found in [21].

The main difference between this configuration of the BFP experiment and those reported previously [7, 11–13, 15] is the positioning and tracking of the pipette: the RMP position was inferred from controlled constrained movement of a piezo translator in earlier work, while our experiments relied on free mobility of the probe. This approach gave us flexibility to work with experimental conditions we could not control (such as the distribution and localization of the beads across the axonal network) and to perform manipulations in the direction and extent we needed. Tracking the pipette and the bead in the same frame also removes the need to synchronize the piezo translator feedback with the video acquisition device.

### Basic approach for analysing good quality recordings

**Fig. 3:**
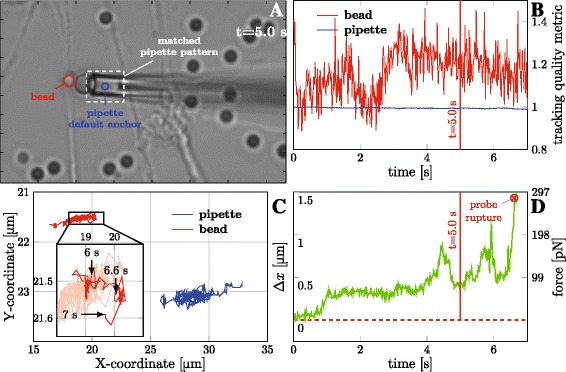
Outputs of quick analysis. Results of simple case analysis of good quality video. **a** Frame from the video, t=5.0 s, contrast is sufficiently high and stable. The bead is marked by the *red ring*, the point tracked on the pipette in *blue*, the pipette tip pattern outlined by the *white dashed box*. **b** Tracking quality metrics of the bead (*red*) and correlation coefficient of the pipette (*blue*). Note that the bead detection quality is very volatile as compared to the pipette. **c** Tracks of the bead centre (*red*) and the pipette anchor point (*blue*) during the experiment time course. In the inset, part of the bead track is magnified; the last second of the path, delimited by the time points 6 s and 7 s, is highlighted (rest is faded). The time point 6.6 s denotes the probe rupture—see panel D. Fluctuations of the bead along the Y-coordinate (perpendicular to the pipette movements) are of order 10 nm. **d** Deformation of the RBC (*Δ* *x*) and the force exerted by the probe during the experiment. *Red dashed line* indicates the zero force, the red mark indicates a separation of the RBC and bead. Total computational CPU time 105 s. The video file used for this analysis, *basic.avi*, is included in the program repository

Figure 3 a shows a screenshot of the video overlaid with the tracking results, the bead delineated in red, the reference point (anchor) on the pipette in blue. In this example, the analysed video was 456 frames long (7.0 s at 65 fps), and the whole interval was treated in a single run. After opening the video, the bead was click-selected, and a tight delineating rectangle was drawn around the pipette tip (white dashed in Fig. 3 a) to delimit the pattern for matching. The tracking procedure could be started immediately.

The panel in Fig. 3 b shows the tracking metrics, with the blue line corresponding to the pipette correlation *M*_*p*_, and the red line to the bead metric *M*_*b*_. Both indicators remain sufficiently stable and elevated during the analysis. While the bead metric tends to be more volatile (which is true also in general), the robustness of the pipette correlation is evident, with the correlation coefficient $M_{p}\gtrsim 0.97$ during the whole interval.

Their tracked positions behave in an orderly manner, as can be seen in Fig. 3 c; no rapid changes in position are present, neither for the bead nor for the pipette anchor. The inset in Fig. 3 c magnifies the track of the bead; for the sake of clarity, only the last second of the track is highlighted (the rest is faded). In the panel, the bead moves predominantly along the x-coordinate, reacting to the movements of the pipette. The bead displacements along the y-coordinate are very limited, of the order of ∼10 nm between consecutive frames, corresponding mostly to the uncertainty of bead-tracking method. Strong metrics and smooth trajectories confirm that the tracking was stable during the analysis and the basic procedure was sufficient.

The time course of RBC deformation *Δ* *x*(*t*) in Fig. 3 d shows gradual changes, with limited and stable variance in time. The red mark indicates the time point at which the probe separated from the bead. We calibrated the probe in two steps: (1) we measured its geometry and calculated the stiffness (see Eq. 1), $k= {198}\frac {\text {pN}}{\upmu \mathrm {m}}$; (2) we identified the frame with the reference distance *x*_0_—the frame of initial contact of RBC with the bead, where the RBC becomes slightly deformed by adhesive forces, but no load is applied (*F*=0). Calibrated values of the measured force could then be obtained. To estimate the uncertainty of the force measurement, we determined the running variance for the force data using a 40-frame window [22]. The standard deviation of force was $F\lesssim 10\,\mathrm {p} \mathrm {N}$ and was independent on the current force magnitude. The measurement uncertainties are systematically discussed in the section Advantages, disadvantages and improvements.

The user has the option to ignore the particular geometry of the current probe and perform a force calculation using pre-defined values. In such case, the information concerning the RBC deformation *Δ* *x* is still clearly valid, but the RBC stiffness $\tilde {k}$ is merely generic and the calculated force $\tilde {F}$ gives only information about the time course, but not the correct magnitude, of the load force.

Additional file 1 is a demonstration video that shows how the analysis was performed in the GUI.

### Advanced options for analysing more complex recordings

**Interrupted tracking:** In many recordings, the stability of focus and the general quality of the video can become sub-optimal; this impairs the detection and may lead to a fatal tracking failure. The user can use the information provided by initial contrast analysis and identify problematic time intervals before starting the tracking procedure. The tool provides features to exclude such intervals from tracking analysis and to link in chain the remaining parts of the recording (*b* in Fig. 2).

**Fig. 4:**
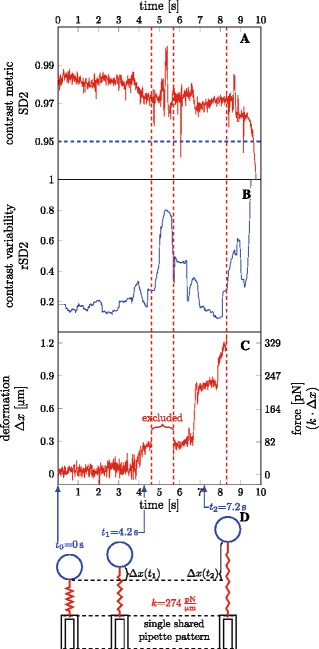
Interrupted tracking. The video could be tracked using a single set of settings, but a gap had to be introduced. **a** Time course of contrast, SD2 of each video frame. No significant drop below the threshold (95%) is observed. **b** Running standard deviation of the contrast metric (rSD2). It defines how variable is the contrast locally. A significant peak can be seen around t=5 s and after t=8 s. This points to intervals of changing focus, which should be treated carefully. **c** The resulting deformation and force. A gap is introduced around t=5 s to avoid tracking failure and again after t=8 s. Because the focus reverts to the previous state in the second tracked interval, we can use the same pipette pattern and the same reference frame, thus the forces of both intervals are fully compatible, with identical calibration. **d** Illustration of BFPtool’s internal treatment of data. On the left, the probe under no load, RBC undeformed (*x*_0_,*Δ* *x*=0). The stiffness of the probe is calculated after the geometrical parameters of the probe (R _*c*_, R_0_, R _*p*_) are measured, yielding $k=274 \frac {\text {pN}}{{\upmu }\text {m}}$. The RBC acts as a spring in the probe. On the right, the probe at any later time, at 4.2 s and 7.2 s as examples. Note the same pipette pattern is shared by both intervals. The RBC is deformed, the deformation corresponds to the change of distance between the bead centre and the pipette anchor, *Δ* *x*(*t*)=*x*(*t*)−*x*_0_, as illustrated. The force is then calculated as *F*(*t*)=*k*·*Δ* *x*(*t*). Total computational CPU time 152 s. The video file used for this analysis, *interrupted.avi*, is included in the program repository

As can be seen in Fig. 4 a, the SD2 contrast metric declines by the end of the video, eventually dropping below the threshold of 0.95 marked by the blue dashed line. This suggests a change in focus of the video, or a major change in the field (e.g. the pipette is removed). The tracking is therefore stopped before this decline begins, as delimited by the red dashed line after *t*=8 s.

The examination of the SD2 metric is not always sufficient to determine problematic interval; another useful indicator is rSD2, representing local (in time) contrast variability. The tracking often fails in intervals of highly variable contrast due to changes in pipette tip intensity profile. Figure 4 b shows rSD2 of the experiment; the peak around *t*=5 s clearly indicates a problematic interval of high contrast variability, which needs to be excluded from tracking. The tracking was therefore split into two intervals, introducing a gap around *t*=5 s. Both intervals were fully compatible, matching the same pipette pattern and using the same frame of reference distance *x*_0_, yielding identically calibrated force. The force time course is shown in Fig. 4 c, with the apparent gap.

Figure 4 d illustrates the physical concept behind the program. The graphic at the time *t*_0_=0 s illustrates the probe with a bead attached and no load applied; under such circumstances, the distance between the pipette anchor and the bead corresponds to the reference distance *x*_0_. The user has to choose the frame *t*_0_ (from the set of frames selected for analysis), which will be used by the program to extract *x*_0_. To calibrate the probe, parameters determining the probe stiffness (Eq. 1) must be provided; $k = {274}\frac {\mathrm {p} \mathrm {N}}{\upmu \mathrm {m}}$ in case of the experiment presented in Fig. 4. At any other time *t*<*t*_0_ or *t*>*t*_0_, the program measures the immediate distance of the pipette anchor and the bead, *x*(*t*); the difference between *x*(*t*) and the reference *x*_0_ corresponds to the RBC deformation. For the example in Fig. 4, $\Delta x(t_{1})~{=}~x(t_{1})-x_{0}|_{t_{1}\phantom {\dot {i}\!}={4.2}\mathrm {s}}=0.3\,\upmu $m, and the force applied by the probe at the time is $\phantom {\dot {i}\!}F(t_{1})~{=~}k\cdot \Delta x(t_{1})|_{t_{1}={4.2}\mathrm {s}}={82}\,\text {pN}$. The experiment in Fig. 4 can be treated by simple exclusion of the problematic interval, because the pipette pattern can be reused in the following interval, i.e. the focus recovers and the same pattern can be matched. Additional file 2 demonstrates how the analysis was performed in the GUI.

**Fig. 5:**
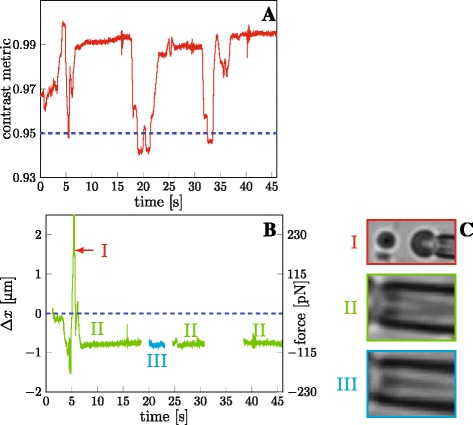
Multi interval tracking. The video had to be split into several intervals due to faltering focus. **a** Time course of contrast, SD2 of each video frame; normalized by the maximal value. *Blue dashed line* marks the warning threshold, 5% drop from the maximum. **b** Tracking was divided into four intervals. The peak marked by the label (I) is a result of separation of pipette and the bead, as shown in panel C-I. Intervals of measured deformation marked in green and labelled (II) are mutually compatible, using the same pipette pattern shown in panel C-II and the same reference distance *x*_0_ — the force calibration is identical. The focus during the interval of data in cyan, labelled (III), was locally stable, and could be tracked using local pipette pattern, before the focus reverted to the original state; the pipette pattern is shown at the panel C-III, clearly different from the pattern C-II. The calibration of the cyan interval was set manually to correspond to the final values of preceding interval. Total computational CPU time 432 s. The video file used for this analysis, *multiinterval.avi*, is included in the program repository

**Multi-interval tracking:** In the previous section, we described how intervals of low contrast SD2 metric should be excluded. In some situations, however, they can be treated differently. Figure 5 a shows one brief and two significant dips in contrast. The decrease does not necessarily mean a change in focus; the first brief dip around time *t*=5 s, marked by label (I) in Fig. 5 b, is a result of probe separation and movement of part of the pipette out of the field (shown in Fig. 5 c-I), which decreases the SD2 metric. The tracking did not fail during the separation; as long as the pipette tip and the bead remain in the field and are recognized by the program, it is not necessary to introduce a gap into tracking, the algorithm can handle such a situation. Despite the bead-pipette tip anchor distance *x*(*t*) having been successfully obtained, it is clear that the peak (I) has no physical meaning. As a general side note, any peak of this type appearing in the extension measurement *Δ* *x*(*t*) should be treated with caution.

Figure 5 b shows the obtained time course of extension *Δ* *x*(*t*), which has three gaps, around times *t*=20 s and *t*=35 s; those are intervals of changing focus, and could not be tracked. Intervals in green, marked by the label (II), are not contiguous, but were tracked using an identical pipette pattern (shown in Fig. 5 c-II) and one common reference distance $x_{0}^{\text {(II)}}|_{t=1.2 \mathrm {s}}$, the focus always eventually recovered. As such, the forces read in all the three segments of (II) are mutually compatible and comparable in terms of value. This it the approach used in Fig. 4.

A different case is the interval marked in cyan, (III): it is a semi-stable period, during which the pipette could be tracked using a locally selected pattern (shown in Fig. 5 c-III) and local reference distance $x_{0}^{\text {(III)}}|_{t=20.0\mathrm {s}}$. Selecting a local pipette pattern and the $x_{0}^{\text {(III)}}$ reference distance can introduce an incompatibility of the obtained force with results of other intervals, if not done very carefully. A frame containing the RBC under no load may not even exist (as in case of Fig. 5 b–III) in some intervals. For the shown (cyan) data, an arbitrary constant was added to the interval results, so that its beginning is aligned to the final force of the preceding interval (II), because the RMP does not move in the meantime, according to the video. Additional file 3 demonstrates how the analysis was performed in the GUI.

### Advantages, disadvantages and improvements

To assess the computational time required for the analysis, the program was tested using Matlab’s profiling tool. The results corresponding to each of the three examples (Figs. 3, 4 and 5) are presented in Additional file 4. Considering the typical length of individual recordings, ranging from minutes in cell manipulation experiments up to 30 min in molecular-bond spectroscopy experiments, the processing time on current desktop machines or laptops is not prohibitive for the user. The processing rate on a low-end office computer (with a 2-core 1.30 GHz CPU) is ∼5 frames per second; for a recording of 5 min at 65 fps, the total computation time is ∼1 h (see Additional file 4 for details).

The tracking is the essential part of the tool. Because *TrackBead* and *TrackPipette* methods are called sequentially for each interval, each analysed frame has to be read twice. The inefficiency could be removed by inserting an intermediate function, which would read each frame only once and provide it (in read-only mode) to the tracking methods, cutting the number of read frames by the factor of two but resulting in increased need for management of returned and forwarded data and number of function calls, making the code more difficult to maintain and debug. Redesigning this part of the program would somewhat improve the computation speed, but would require significant changes to already tested code.

The analysis becomes substantially more demanding for recordings of low quality, containing frames for which the tracking quality metrics do not exceed the preset thresholds. In such cases, the program attempts to refine the detection by repeating the analysis on a larger sub-field of the subthreshold frame, with dilated or eroded pipette pattern, higher edge sensitivity etc. These methods generally increase the value of the detection metric and help to avoid tracking failures. Pipette dilation/erosion method, for example, can improve the correlation metric by 10% for initial values *M*_*p*_≈0.5. The methods however consume considerable amount of computational time, particularly in case of the pipette.

In order to obtain an estimate of the general tracking precision in a typical recording, we have analysed the variance of outputs. The uncertainty of the RBC deformation measurement *ε*_*Δ*_, and the corresponding force uncertainty *ε*_*F*_, was calculated as a running standard deviation on intervals of static equilibrium (plateaus), while the intervals containing manipulations were avoided. The results were largely independent of the used size of the running window (between 10 and 100 frames, for 65 fps videos). For the experiment presented in Fig. 3, *ε*_*Δ* ≈_50 nm, which corresponds to *ε*_*F*_ ≈ 10 pN; for Fig. 5, *ε*_*Δ*_ ≈ 35 nm, which corresponds to *ε*_*F*_ ≈ 4 pN; for Fig. 4, *ε*_*Δ*_ ≈ 44 nm and *ε*_*F*_ ≈ 12 pN. These results are the upper bounds of the tracking method uncertainty, as it is not possible to completely eliminate all pipette movements from the error analysis.

These values are consistent with the anticipated precision of the tracking method (see Fig. 3). The pattern matching uses more pixels for the calculation, and pipette position is further improved by interpolation by a second degree polynomial, which makes the pipette detection one order of magnitude more precise that the bead detection. As long as the pipette metric remains strong, the error in the RBC deformation measurement can be largely attributed to the bead tracking. The precision of bead tracking (using Gaussian blob fitting) reported in literature varies over one order of magnitude, 5–30 nm (5 nm in [7, 15, 17], and 30 nm in [14]). The method applied in our tool might have slightly lower performance at the trade-off of higher robustness. It performs well under complex situation of faltering contrast and more importantly during considerable obscuring of the bead—more that half of the edge of bead in some situations. It is not dependent on the bead intensity profile (i.e. it does not need to be Gaussian).

It should be noted that the calculated value of the load force *F* is affected by a systematic error arising from the evaluation of the probe stiffness *k*. If the probe geometry is measured directly from the video, the limited resolution of the optical microscope leads to the relative error of roughly *δ* *k* ≈ 20*%*, for the typical values of *R*_0_ = 2.5 *μ*m, *R*_*p*_ = 1.0 *μ*m, *R*_*c*_ = 0.75 *μ*m and *Δ* *P* = 200 Pa, and the measurement uncertainty 0.2 *μ*m for each diameter (i.e., 0.1 *μ*m for each radius); the uncertainty in aspiration pressure, 5Pa, does not contribute significantly. This estimate of *δ* *k* is comparable with the value of 14% reported by [17]. The tool calculates and provides the magnitude of this geometry-dependent uncertainty to the user during the calibration step. Note that in case the video was captured with sub-optimal resolution, this systematic error may further increase. Another major source of systematic error is the reference distance *x*_0_. It can be difficult to precisely localize an optimal calibration frame; an improper choice $\tilde {x}_{0}{\neq }x_{0}$ shifts all the obtained values of deformation (and force) by a constant.

Last, but definitely not least, is a systematic error stemming from the linear axial approximation of the probe. The more the probe is off axis (i.e. the pipette axis, the centre of the RBC and the bead are not in line), the less accurate the transformation based on Eq. 1 becomes. This can be slightly improved by manual selection of the pipette anchor so that it is in line with the RBC and bead centres, although it may mean putting it off the pipette axis. The linear approximation (allowing to treat the RBC as a spring) is valid up to *Δ* *x*≤ 0.5 *μ*m (or error <5 % for *Δ* *x*≤ 0.3 *μ*m as reported by [17]), for larger deformations, it tends to slightly over-estimate the force. It would improve the program if the full and more general equation of geometry-force relation were implemented, incorporating non-linear terms and the influence of deviations from the axis. While a non-linear axial equation was carefully derived by Simson in [17], it is complex and its numerical solution iterative, making its implementation non-trivial.

The influence of axial deviations in BFP has not yet been studied. A detailed understanding of the force-deformation relation for non-axial probe would make BFP more versatile in applications to cell interfaces (as in our experiments) and as a tool for biophysical manipulations. It would however require to keep track of the angle of deviation, the general RBC shape projection (not only extension) and other possible geometrical descriptors, thus increasing the demands on tracking algorithms in terms of computational cost and robustness.

## Conclusion

The present *BFPTool* application is (to our knowledge) the first freely available software for automated analysis of BFP image data and of the exerted force. It has been made with the end-user in mind: a comprehensive and comprehensible GUI was implemented, permitting a fast-track use supplemented by optional advanced functions, running extensive verifications and generating reports and warnings. We made the best efforts to avoid crashes and exceptions and to instead provide the user with assertions and reports, with limited impact on performance.

The program offers tools to selectively analyse recorded videos of BFP experiments. The user is guided in several ways to exclude faulty segments of the recording and concatenate the remaining parts in an optimal manner, to obtain coherent results. Many internal tracking parameters can be finely tuned to fit the particular experiment. The geometry of the experiment can be measured directly using embedded methods.

The tool performs the tracking of the components of BFP apparatus and analyses the outcomes to calculate the time course of applied force. Both the detected tracks (and their corresponding tracking fidelity metrics) and the force can be visualized and fitted by standard functions (linear, exponential, plateau detection). The final results can be exported in form of multimedia or data, while the ongoing work can be saved and loaded at later time or on a different computer.

A novelty brought by the tool is the capacity to track the pipette, which was localized only indirectly in previously reported BFP experiments. The pipette tracking from the image data is sufficiently precise so as not to affect the technique’s accuracy. The possibility to precisely track the pipette while freely moving the probe (not relying on limited-range piezo translators) opens the BFP technique to more versatile use. In addition, many labs are equipped with micromanipulators but do not have a piezo stage. The present approach allows them to use the technique directly without further investment.

The software tool complements the BFP technique to form a practical and accessible instrument for biophysical applications—interface and membrane experiments, and micro-manipulations of cells or vesicles. It can be used to induce cellular movement in a soft and controlled manner, or to measure adhesion, tension or rigidity, applying calibrated forces with uncertainty at the order of 10 pN. We hope that this software will help to develop and popularize the BFP technique, while simplifying work for those who already use it.

## Availability and requirements

**Project name:** BFPTool **Project homepage:** BFPtool at GitHub, https://github.com/smitdaniel/BFPtool **Operating system:** cross-platform under Matlab **Programming language:** Matlab **Requirements:** Matlab 2015a, Image Processing Toolbox **License:** GPL 3 **Any restrictions to use by non-academics:** none

## Endnote

^1^ Tilde symbol denotes an estimate of the given quality obtained without proper calibration.

## Additional files

Additional file 1 This video describes how to set up the simple analysis without interruption, presented in Fig. 3. (MKV 1730 kb)

Additional file 2 This video describes how to set up the analysis with an excluded time interval, presented in Fig. 4. (MKV 1730 kb)

Additional file 3 This video describes how to set up the analysis broken into several intervals with different pipette template patterns, presented in Fig. 5. (MKV 3700 kb)

Additional file 4 The results of profiling of the BFP runs, presented in Figs. 3, 4 and 5, demonstrating the distribution of computational demands. (PDF 58.7 kb)

## Abbreviations

BFP: Biomembrane force probe
fps: Frames per second
GUI: Graphical user interface
IA: Image analysis
I/O: Input and output
RBC: Red blood cell
RMP: RBC-mounted pipette
rSD2: Running standard deviation of SD2 contrast metric
SD2: Two-dimensional standard deviation
