# Supplementary material for: BFPTool: a software tool for analysis of Biomembrane Force Probe experiments
Source: BMC Biophys. 2017 Feb 13;10:2. doi: 10.1186/s13628-016-0033-2 (PMC5304404; doi:10.1186/s13628-016-0033-2)
Supplement: Additional file 4 — The results of profiling of the BFP runs, presented in Figs. 3, 4 and 5, demonstrating the distribution of computational demands. (PDF 58.7 kb) [file 13628_2016_33_MOESM4_ESM.pdf]

BFPTool runtime profiling results
for the analyses described in main text

The analyses of BFP experiments presented in Figs. 3, 4 and 5 of the main text were profiled to assess the distribution of processing time requirements. All the tests were performed on a AMD Athlon II X2 Neo K325 Dual-Core 1.30 GHz CPU and 2.7 GiB RAM. The time needed to process a video was around 20 s per 100 frames, corresponding to 13 min of processing time per minute of recording at 65 fps.

The profiling shows that the pipette tracking is more computationally demanding than the bead tracking, depending on the pipette pattern size and number of calls to corrective methods (i.e. detection threshold settings). It also shows that processing of hundreds to thousands of frames has about the same efficiency for TIFF and AVI files, despite the much larger size of the TIFF files. TIFF files larger than 4 GiB are not generated properly by the most popular program ImageJ, and at the time not supported by the BFPtool.

Method	AVI format	TIFF format
normxcorr2 (pipette)	41 % (43 s)	51 % (43 s)
imfindcircles (bead)	21 % (22 s)	26 % (22 s)
video reading	27 % (28 s)	6 % (5 s)
waitbar	6 % (6 s)	8 % (7 s)
total time	105 s	84 s

Table 1: Corresponding to Fig. 3, tracked 456 frames (t=7.0 s at 65 fps).

Method	AVI format	TIFF format
normxcorr2 (pipette)	49 % (75 s)	61 % (79 s)
imfindcircles (bead)	18 % (27 s)	21 % (27 s)
video reading	21 % (32 s)	5 % (6 s)
waitbar	7 % (11 s)	8 % (10 s)
total time	152 s	130 s

Table 2: Corresponding to Fig. 4, tracked 510 frames (t=7.8 s at 65 fps).

Method	AVI format	TIFF format
normxcorr2 (pipette)	34 % (148 s)	34 % (151 s)
imfindcircles (bead)	25 % (110 s)	24 % (109 s)
video reading	33 % (141 s)	34 % (153 s)
waitbar	4 % (19 s)	4 % (18 s)
total time	432 s	447 s

Table 3: Corresponding to Fig. 5, tracked 2300 frames (t=35.4 s at 65 fps).
